# Growth and adaptation of Zika virus in mammalian and mosquito cells

**DOI:** 10.1371/journal.pntd.0006880

**Published:** 2018-11-12

**Authors:** Lindsey A. Moser, Brendan T. Boylan, Fernando R. Moreira, Laurel J. Myers, Emma L. Svenson, Nadia B. Fedorova, Brett E. Pickett, Kristen A. Bernard

**Affiliations:** 1 Department of Pathobiological Sciences, School of Veterinary Medicine, University of Wisconsin-Madison, Madison, WI, United States of America; 2 Department of Infectious Diseases, J. Craig Venter Institute, Rockville, MD, United States of America; Centro de Pesquisas René Rachou, BRAZIL

## Abstract

The recent emergence of Zika virus (ZIKV) in the Americas coincident with increased caseloads of microcephalic infants and Guillain-Barre syndrome has prompted a flurry of research on ZIKV. Much of the research is difficult to compare or repeat because individual laboratories use different virus isolates, growth conditions, and quantitative assays. Here we obtained three readily available contemporary ZIKV isolates and the prototype Ugandan isolate. We generated stocks of each on Vero mammalian cells (ZIKV^mam^) and C6/36 mosquito cells (ZIKV^mos^), determined titers by different assays side-by-side, compared growth characteristics using one-step and multi-step growth curves on Vero and C6/36 cells, and examined plaque phenotype. ZIKV titers consistently peaked earlier on Vero cells than on C6/36 cells. Contemporary ZIKV isolates reached peak titer most quickly in a multi-step growth curve when the amplifying cell line was the same as the titering cell line (e.g., ZIKV^mam^ titered on Vero cells). Growth of ZIKV^mam^ on mosquito cells was particularly delayed. These data suggest that the ability to infect and/or replicate in insect cells is limited after growth in mammalian cells. In addition, ZIKV^mos^ typically had smaller, more homogenous plaques than ZIKV^mam^ in a standard plaque assay. We hypothesized that the plaque size difference represented early adaptation to growth in mammalian cells. We plaque purified representative-sized plaques from ZIKV^mos^ and ZIKV^mam^. ZIKV^mos^ isolates maintained the initial phenotype while plaques from ZIKV^mam^ isolates became larger with passaging. Our results underscore the importance of the cells used to produce viral stocks and the potential for adaptation with minimal cell passages. In addition, these studies provide a foundation to compare current and emerging ZIKV isolates *in vitro* and *in vivo*.

## Author summary

The ZIKV scientific field has greatly expanded since the emergence of ZIKV in South and Central America, but a comprehensive comparison of the assays used to examine the phenotypic and replicative properties of ZIKV is limited in the literature. The influence of host, whether insect or mammalian, on viral production and infection has not been thoroughly examined for ZIKV. Additionally, a number of different assays are used in the literature to examine ZIKV, but how results compare across assays and between laboratories is unclear. We provide a detailed *in vitro* characterization of growth parameters in both mosquito and mammalian cells for one reference and three contemporary ZIKV isolates. These studies provide the basis for other researchers to compare results and to build on for future animal and cell culture studies with current and emerging ZIKV isolates.

## Introduction

Zika virus (ZIKV) is a mosquito-borne virus in the genus *Flavivirus*, which includes many arthropod-borne viruses (arboviruses) such as dengue virus (DENV) and West Nile virus (WNV). It was originally isolated in 1947 from a sentinel macaque in Uganda and was subsequently found throughout Africa and Asia with minimal reports of disease [1–6]. In 2013, ZIKV emerged in French Polynesia, resulting in the infection of 66% of the island’s population as well as a 45-fold increase in the incidence of the neurological disease Guillian-Barre syndrome and increased risk of microcephaly in newborns [7–9]. ZIKV emerged explosively in Brazil in 2015 [10] with estimates of over 1.5 million infections [11] and quickly spread throughout South and Central America and the Caribbean with 84 countries reporting local mosquito transmission (WHO situation report, 10 March 2017). Sporadic autochthonous transmission was documented as far north as Florida and Texas [12, 13]. In addition, travel-acquired cases were documented throughout the Americas and Europe (Centers for Disease Control and Prevention, European Centre for Disease Control and Prevention). The extensive geographic range of ZIKV and severe congenital abnormalities in infants born to ZIKV-infected mothers have resulted in a worldwide public health concern.

ZIKV is primarily transmitted through the bite of infected mosquitoes [14, 15] although congenital and sexual transmission also occur [16–21]. *Aedes* species mosquitoes, particularly *A*. *aegypti* and *A*. *albopictus*, are important vectors of ZIKV transmission [6, 22–26]. The alternating passage through disparate hosts (arthropod and mammalian) likely contributes to the conserved consensus sequence of arboviruses in nature in comparison to other RNA viruses [27, 28]. Transmission and replication in vertebrates and invertebrates place varied evolutionary pressures on arboviruses [29, 30]. According to the trade-off hypothesis, an arbovirus population is maintained at a sub-optimal fitness level in each species, in theory allowing it to efficiently replicate in both hosts [31]. Selection for better replication by repeated passage of the virus in one species is predicted to decrease fitness in the alternate host. Because the high mutation rates of RNA viruses allow viruses to expand into new niches, this suggests an intrinsic limitation of arboviruses to adapt to new environments. Many studies suggest that this is not true, however, likely due to the fact that arboviruses exist not as single genetic species, but as population swarms. These viral quasispecies maintain a conserved consensus genome, but there are large numbers of mutations found at low frequencies. Many of these mutations have either minimal phenotypic effects or are deleterious to the virus; however, selection pressures on the mutant swarm can select for advantageous mutations that contribute to phenotypic changes [32–35]. In this way, arboviruses can successfully adapt and respond to new environments.

In this study, we compared commonly used assays to characterize the phenotype of four readily available ZIKV isolates in mammalian and mosquito cells. ZIKV stocks were produced by a single passage on mammalian or mosquito cells. We compared the effect of the deriving cell line on subsequent growth and spread in homologous and heterologous cells. We showed that mammalian cell-derived and insect cell-derived ZIKV differ in infection and growth kinetics. We further investigated a ZIKV isolate that exhibited marked differences in plaque phenotype dependent on growth in insect or mammalian cells, and demonstrated growth restriction in an insect, but not mammalian, cell line. These studies provide a foundation to characterize and compare current and emerging ZIKV isolates.

## Methods

### Cell lines and virus stocks

Vero African green monkey kidney cells (CCL-81; ATCC) were grown in complete medium (minimal essential medium (MEM; Sigma) with 10% fetal bovine serum (FBS, Atlanta Biologicals) plus 1X non-essential amino acids [NEAA, Sigma]) at 37°C in 5% CO_2_. *Aedes albopictus* mosquito C6/36 cells (CRL-1660; ATCC) were grown in complete medium (MEM with 10% FBS and 1X NEAA) at 28°C in 5% CO_2_.

ZIKV isolates ZIKV/*Homo sapiens*/PAN/CDC-259249_V1-V3/2015 (PAN), ZIKV/*Homo sapiens*/PRI/PRVA BC59/2015 (PRV), ZIKV/*Homo sapiens*/COL/FLR/2015 (FLR), and ZIKV/*Macaca mulatta*/UGA/MR-766_SM150-V8/1947 (MR-766) were received from BEI Resources. Isolate isolation and passage history is listed in Table 1. The seed virus from BEI was used to generate virus stocks from Vero mammalian cells (ZIKV^mam^) and C6/36 mosquito (ZIKV^mos^) cells. Briefly, Vero or C6/36 cells were inoculated at a multiplicity of infection (MOI) 0.01 in virus diluent (MEM + 1% FBS). The infection proceeded for 1 hr at 37°C (Vero) or 28°C (C6/36). Complete medium was added, and the cells were incubated at 37°C (Vero) or 28°C (C6/36). An aliquot was collected every 24 hpi, and the cells were monitored daily for the development of cytopathic effect (CPE) compared to mock-inoculated control cells. CPE was apparent when ZIKV isolates were grown on Vero cells, and culture medium was harvested at 3 dpi for PAN, PRV, and MR-766 or at 5 dpi for FLR. No CPE was evident when ZIKV isolates were grown on C6/36 cells; thus, culture medium was harvested from all isolates at 6 dpi when C6/36 virus titers peaked. Virus stocks were clarified by centrifuging the culture medium at 15000 x g for 10 min at 4°C. Virus growth samples and final stocks were titered by plaque assay (PA) as described below. Stocks were stored in single use aliquots at -80°C.

**Table 1:** ZIKV isolates used in this study.

ZIKV Isolate Name	Isolation Location	Isolation Host	BEI Reference Number	GenBank Accession Number^1^	Passage History^2^
ZIKV/*Homo sapiens*/PAN/CDC-259249_V1-V3/2015	Panama	*Homo sapiens*	NR-50219	KX156775	Passaged 4 times in Vero cells
ZIKV/*Homo sapiens*/PRI/PRVABC59/2015	Puerto Rico	*Homo sapiens*	NR-50240	KX087101	Passaged 5 times in Vero cells
ZIKV/*Homo sapiens*/COL/FLR/2015	Colombia	*Homo sapiens*	NR-50183	KX087102	Passaged 3 times in C6/36 cells
ZIKV/*Macaca mulatta*/UGA/MR-766_SM150-V8/1947	Uganda	*Macaca mulatta*	NR-50065	KU963573	Passaged 150 times in sucking mice and 8 times in Vero cells

^1^Accession number refers to the sequence of the lot of virus received. PRV has been previously sequenced under accession number KU501215, FLR under accession number KU820897, and MR-766 under accession numbers AY632535 and DQ859059.

^2^Passage history refers to virus history at receipt from BEI Resources.

### Plaque assay

Samples were diluted sequentially 10-fold in virus diluent. Diluted sample was added in duplicate to confluent Vero cell monolayers, and the plates were incubated for 1 hr at 37°C, 5% CO_2_. Monolayers were overlaid with 3 ml 0.6% oxoid agar (Thermo Fisher) in overlay medium (MEM with 5% FBS, 1X NEAA, and 1X penicillin-streptomycin (Gibco)). The plates were incubated at 37°C for 3 days for PRV and PAN or 4 days for FLR and MR-766. Monolayers were then stained by adding 2 ml of staining overlay (0.6% oxoid agar and 82.5 mg/L neutral red (Sigma) in overlay medium) to each well. Plates were incubated for 24 hours at 37°C, and virus titer was determined based on the number of plaques in each well.

### Real-time quantitative (q) RT-PCR assay

RNA from 100 μL clarified virus supernatant was extracted using an RNeasy kit (Qiagen) and eluted in 50 μL RNAse-free water. The concentration of virus (genomic equivalents [GE]/ml supernatant) for each stock was determined by qRT-PCR using ABI TaqMan RNA-to-C_T_1-step Kit (Life Technologies) according to the manufacturer’s instructions. Primers and probe were based on those published by Lanciotti *et al* [36], modified to recognize the E gene of contemporary and reference ZIKV isolates (ZIKV-1086F: YCGYTGCCCAACACAAG; ZIKV 1162R: CCACTAAYGTTCTTTTGCAGACAT; ZIKV-probe: Fam-AGCCTACCTTGACAAGCAATCAGACACTCAA-Tamra). ZIKV-PRV^mam^ RNA concentration was determined by nanodrop (ThermoFisher), and the number of GE was calculated and used for a standard curve (10^0^−10^9^ GE). GE:PFU ratios were determined by dividing the GE concentration by the concentration of infectious virus determined in the PA.

### Fluorescent focus assay (FFA)

Vero or C6/36 cells were grown to confluence in 24-well plates. Cells were inoculated with 10-fold dilutions of ZIKV, incubated for 1 hour at 37°C (Vero cells) or 28°C (C6/36 cells), and overlaid with 0.8% methylcellulose (MP Biomedicals) in complete medium. FFAs on Vero cells and C6/36 cells were set up in parallel, using the same dilutions of sample. Cells were incubated for 4 days (Vero cells) or 6 days (C6/36 cells). The overlay was removed, and cell monolayers were washed twice with PBS and fixed with 10% formalin for 30 minutes. Cells were permeabilized with blocking buffer (0.1% Triton-X 100 (Fisher Scientific) in PBS), blocked with 3% normal goat serum in blocking buffer, and probed with pan flavivirus antibody clone 4G2 (EMD Millipore) diluted 1:1000 in blocking buffer. Monolayers were washed 3 times with PBS and incubated with HRP-conjugated anti-mouse antibody (1:1000 in blocking buffer). Cell monolayers were washed 3 times with PBS, and foci were visualized using True Blue Developing Substrate (Kirkegaard & Perry Lab, Inc.) per manufacturer’s recommendations.

### TCID_50_ assay

Vero cells were grown to confluence in 96-well plates and inoculated with 10-fold dilutions of ZIKV samples. Cells were incubated for 6 days at 37°C. Monolayers were fixed by adding formalin to a final concentration of 5% for 30 minutes. Monolayers were washed twice with PBS, stained with crystal violet (EMD Millipore, 0.2% w/v in 2% methanol) for 10 minutes, and washed twice with tap water. CPE was evaluated visually and compared to mock-inoculated cell monolayers. Virus titer was calculated using the Reed and Muench method [37].

### One-step and multi-step growth curves

ZIKV^mam^ or ZIKV^mos^ was added to Vero cells or C6/36 cells at an MOI of 1 or 3 for one-step growth curves or an MOI of 0.005 for multi-step growth curves. An MOI of 1 was used in one-step growth curves if the titer of the stock virus was too low to obtain an MOI of 3. Quadruplicate wells of a 24-well plate containing either Vero or C6/36 cells were infected at 37°C or 28°C respectively for one hour. The inoculum was removed, and the monolayer was washed three times with virus diluent prior to the addition of complete medium. Vero cells were incubated at 37°C, and C6/36 cells were incubated at 28°C. Aliquots were collected from the supernatant of each well immediately following infection (1 hpi) and then every 24 hours through 5 dpi for Vero cells or through 10 dpi for C6/36 cells. Samples were stored at -80°C until titers could be determined by PA.

### Plaque purification

**Fig 1:**
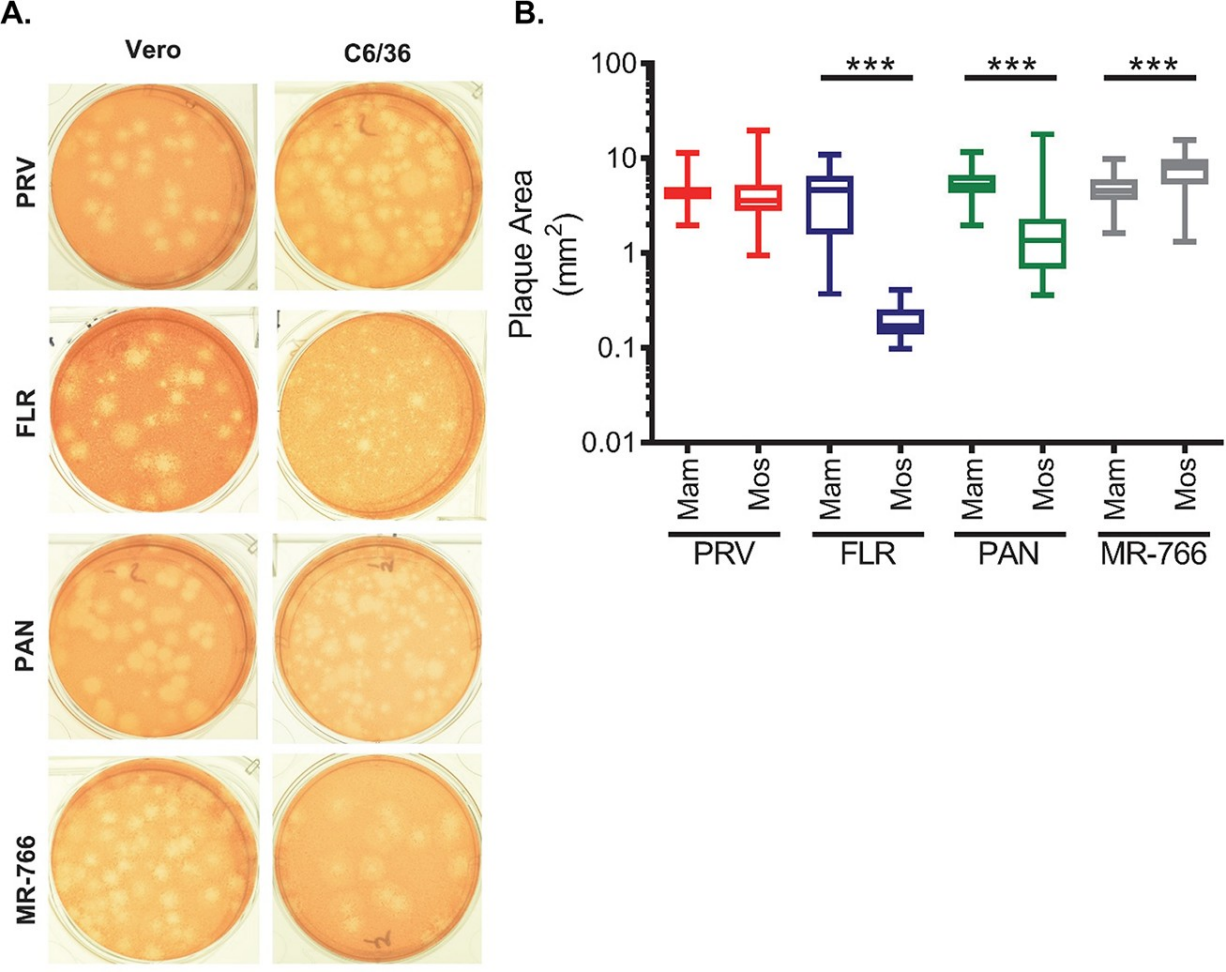
ZIKV isolates grown on mammalian and mosquito cells show phenotypic differences. A) ZIKV-PRV, ZIKV-PAN, ZIKV-FLR, and ZIKV-MR-766 stocks grown on either Vero mammalian or C6/36 mosquito cells were plaqued on Vero cells and visualized with neutral red staining 4–5 days after inoculation. B) The area of 30–50 individual plaques was measured for each isolate of ZIKV using ImageJ.

ZIKV-FLR^mam^ and ZIKV-FLR^mos^ were plaqued on Vero cells as described above. ZIKV-FLR^mam^ produced four sizes of plaques (Fig 1): tiny (L1), small (L2), medium (L3), and large. Two phenotypes were associated with large plaques (L4 and L5). ZIKV-FLR^mos^ produced three plaque sizes: tiny (L1), small (L2), and medium (L3). Three independent clones representing each phenotype class (with the exception of ZIKV-FLR^mos^-L3) were picked with a pipette tip, which was rinsed in 150 μL virus diluent. The plaque was amplified on the same cells from which the virus stock was derived. Virus diluent was inoculated onto Vero or C6/36 cells, and the cells were incubated for 1 hr at 37°C or 28°C respectively. Complete medium was added to each well. Vero cells were incubated at 37°C for 4 days, and C6/36 cells were incubated for 7 days at 28°C. Incubation times were determined as the time at which virus levels began to plateau in the multi-step growth curve. Supernatants were collected, clarified at 12,000 x g for 30 min at 4°C, and plaqued on Vero cells to determine virus titer and plaque phenotype. Clarified supernatants were stored at -80°C. Two additional rounds of plaque purification and amplification were conducted for a total of three rounds. The PA plates were photographed using a Nikon digital camera. The area of 30–50 discrete plaques for each sample was measured using ImageJ (NIH). RNA from the third round virus plaque picks (3 biological clones/condition) was sequenced as described below.

### Genome sequencing and read assembly

RNA samples were sequenced as described in our previous report [38] using the RNA sequence-independent single-primer amplification (SISPA) method [39, 40]. All samples were sequenced using 300 bp paired-end reads on an Illumina MiSeq instrument with a subset of samples sequenced on an Ion Torrent instrument. Read assembly was performed as previously described [38]. Briefly, after reads were deconvoluted and trimmed, the contigs were mapped to the most appropriate ZIKV genome. For sites where the majority of reads disagreed with the sequence from the reference strain, the reference sequence was updated accordingly to improve read mapping in subsequent assemblies. Curated assemblies were validated and annotated with the Viral Genome ORF Reader (VIGOR) version 3 annotation software [41] before submission to GenBank (accession numbers MF574552 to MF574577). Raw data was submitted to the Sequence Read Archive at NCBI under the study accession number SRP162155.

### Data normalization and statistical analysis

All quantitative data were log-transformed and are presented with the mean plus standard deviation. Statistical significance was determined by Student’s t-test using GraphPad Prism and was defined as P<0.05. Significance between a control and an experimental group is indicated as follows: * P<0.05, ** P<0.01, *** P<0.001.

## Results

### ZIKV amplification and quantification

ZIKV seed stocks were amplified by a single passage on mammalian or mosquito cells, resulting in two stocks of each isolate: one grown on Vero cells (ZIKV^mam^) and one grown on C6/36 cells (ZIKV^mos^). The virus stocks were quantified using several common assays on Vero and C6/36 cells (Table 2). The contemporary isolates of ZIKV (ZIKV-PAN, ZIKV-PRV, and ZIKV-FLR) had similar titers when produced on either cell type (titers ≥ 10^7^ PFU/ml by PA on Vero cells); titers of ZIKV^mam^ ranged from equal to 3-fold higher than ZIKV^mos^ for an individual isolate. In contrast, the virus titers of the prototype reference isolate ZIKV-MR-766 were approximately 40-fold higher on mammalian cells than mosquito cells. Similar results were observed when viruses were quantified using a tissue culture infectious dose-50 (TCID_50_) assay; however, the titers by TCID_50_ were 3- to 22-fold lower than by PA, suggesting that the TCID_50_ is less sensitive. We also determined the number of genomic equivalents (GE) by qRT-PCR and calculated the GE:PFU ratio for each stock. The highest GE:PFU ratio of 7.25x10^3^ was observed for ZIKV-PAN^mam^, and ZIKV-PRV^mos^ had the lowest ratio of 5.96x10^2^. The GE:PFU ratios for the other virus stocks were 1-4x10^3^ whether the virus was derived from mammalian or mosquito cells. The ZIKV^mos^ stock had lower GE:PFU ratios compared to the corresponding ZIKV^mam^ stock (1.5- to 7-fold differences). The higher GE:PFU ratio for ZIKV^mam^ may be due to greater amounts of immature particles, inactive particles, and/or release of viral RNA due to CPE into the medium.

**Table 2:** ZIKV titers by assay tested in this study.

ZIKV Isolate	ZIKV Isolate Designation	Cell Line Used to Amplify	Plaque Assay Titer^1^ (PFU/ml)	TCID_50_ Titer^1^ (TCID_50_/ml)	FFA Titer^1^ (FFU/ml)	FFA Titer^2^ (FFU/ml)	GE^3^:PFU Ratio^4^
ZIKV/*Homo sapiens*/PAN/CDC-259249_V1-V3/2015	ZIKV-PAN	Vero	3.5x10^7^	6.3x10^6^	1.6x10^7^	4.0x10^6^	7.25x10^3^
		C6/36	1.3x10^7^	8.4x10^5^	1.1x10^7^	3.5x10^7^	1.02x10^3^
ZIKV/*Homo sapiens*/PRI/PRVABC59/2015	ZIKV-PRV	Vero	9.0x10^7^	6.3x10^6^	2.9x10^7^	1.0x10^7^	2.89x10^3^
		C6/36	2.6x10^7^	2.0x10^6^	2.0x10^7^	2.5x10^7^	5.96x10^2^
ZIKV/*Homo sapiens*/COL/FLR/2015	ZIKV-FLR	Vero	1.3x10^7^	4.3x10^6^	2.1x10^7^	2.3 x10^6^	3.99x10^3^
		C6/36	1.4x10^7^	6.3x10^5^	1.6x10^7^	9.0 x10^7^	2.60x10^3^
ZIKV/*Macaca mulatta*/UGA/MR-766_SM150-V8/1947	ZIKV-MR-766	Vero	6.5x10^7^	2.0x10^7^	4.7x10^7^	1.6x10^8^	3.11x10^3^
		C6/36	1.7x10^6^	9.3x10^4^	4.7x10^5^	1.8x10^6^	9.77x10^2^

^1^Virus was titered on Vero cells.

^2^Virus was titered on C6/36 cells.

^3^Genomic Equivalents (GE)

^4^PFU was determined by plaque assay on Vero cells.

We further characterized the virus stocks by using a focus-forming assay (FFA), which measures all infectious viruses, not just viruses that cause enough cell death to form a visible plaque. The PA and FFA resulted in similar titers (equivalent to less than 4-fold differences) for the same stock of ZIKV, suggesting that there are minimal infectious non-plaque forming viruses in the virus stock populations (Table 2). We also used the FFA to directly compare the ZIKV stock titers on Vero and C6/36 cells in parallel assays (Table 2). We could not use the PA or the TCID_50_ assay, which require cell death, to compare stocks because C6/36 cells do not develop cytopathology with ZIKV infection [42]. For the contemporary isolates, the ZIKV^mam^ titers by FFA on Vero cells were 3- to 9-fold higher than on C6/36 cells; in contrast, the ZIKV^mos^ titers by FFA on Vero cells were equivalent to or 6-fold lower than on C6/36 cells. For the prototype ZIKV-MR-766, the FFA titer on Vero cells was 3- to 4-fold lower than on C6/36 cells for both ZIKV^mam^ and ZIKV^mos^ stocks. These results suggest that the contemporary ZIKV isolates had adapted in just one cell passage to become more infectious for the cell line on which they were derived.

In the process of determining titers, we observed differences in the plaque phenotypes between ZIKV^mam^ and ZIKV^mos^ stocks for the same isolate of ZIKV (Fig 1A). We compared the size profile of each isolate by measuring the area of 30–50 plaques (Fig 1B). ZIKV^mos^ plaques were significantly smaller than ZIKV^mam^ plaques for ZIKV-FLR and ZIKV-PAN; ZIKV-PRV^mos^ showed greater variability in plaque size than ZIKV-PRV^mam^. Tiny plaques dominated the plaque phenotypes for ZIKV-FLR^mos^ (mean size of 0.2 mm^2^) compared to ZIKV-FLR^mam^ (mean size of 4.4 mm^2^). In contrast, ZIKV-MR-766^mos^ plaques were significantly larger than ZIKV-MR-766^mam^. These differences in plaque phenotype suggest that ZIKV is adapting to growth in different cell types after a single passage.

### ZIKV growth in mammalian and mosquito cells

**Fig 2:**
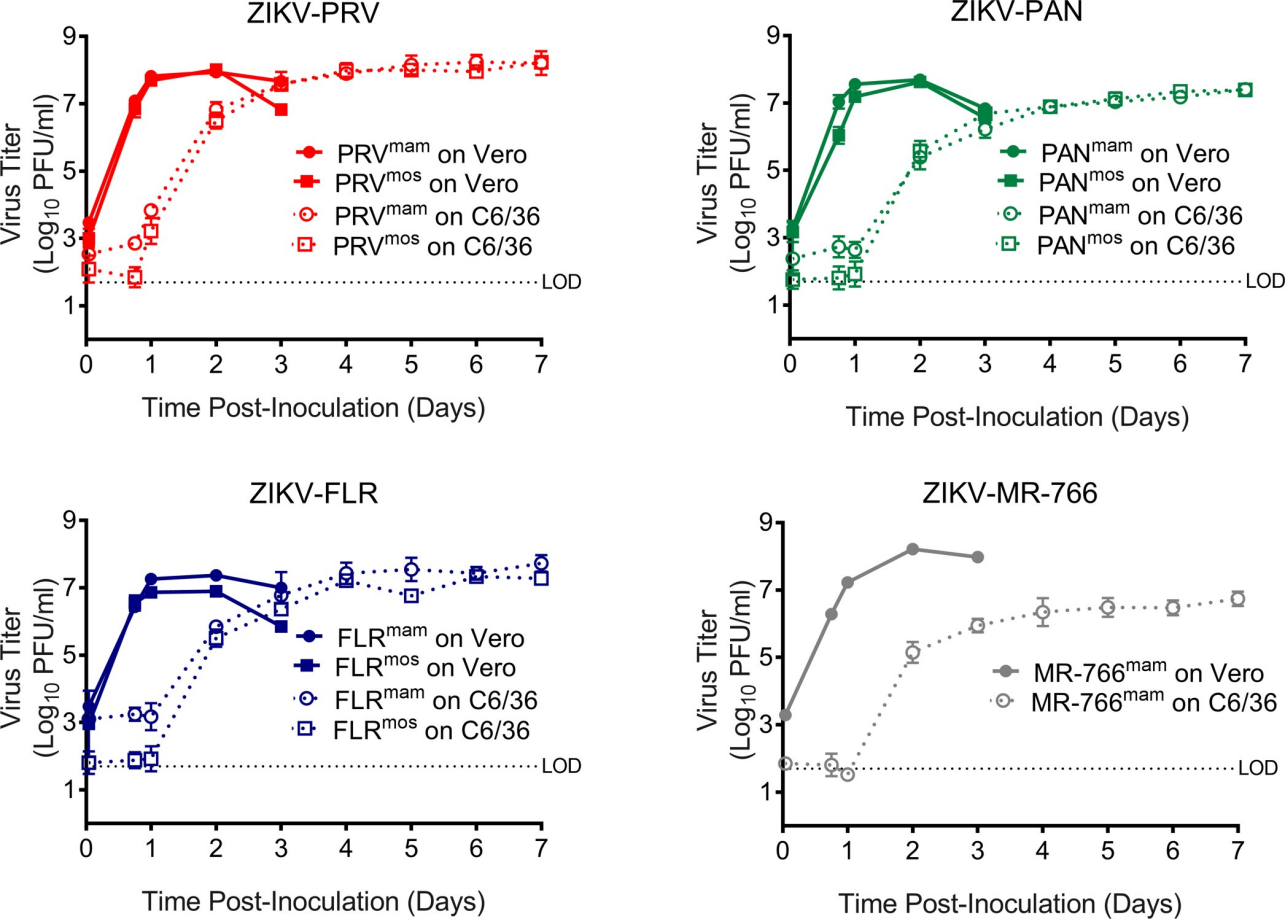
Replication kinetics of single-round infection on Vero or C6/36 cells is similar between ZIKV^mam^ and ZIKV^mos^. Vero or C6/36 cells were inoculated at an MOI 1–3 with ZIKV-PRV, ZIKV-PAN, ZIKV-FLR, and ZIKV-MR-766 grown on either Vero mammalian cells (ZIKV^mam^) or C6/36 mosquito cells (ZIKV^mos^). ZIKV-MR-766^mos^ was omitted due to insufficient titer. Samples were collected at the indicated times and titered by plaque assay on Vero cells. Symbols represent the average, and error bars represent the standard deviation. Some error bars are smaller than the symbol and are not visible.

We conducted one-step and multi-step growth curves on Vero mammalian and C6/36 mosquito cells to examine and compare the replication characteristics of the different ZIKV isolates. For one-step growth curves, Vero and C6/36 cells were inoculated with ZIKV^mam^ and ZIKV^mos^ stocks at a high multiplicity of infection (MOI 1–3). The stock titer of ZIKV-MR-766^mos^ was too low to attain a high MOI; therefore, this sample was omitted from this experiment. Samples of supernatant were collected at various times after inoculation, and virus production was measured by PA. The results are shown for each virus isolate individually in Fig 2, or virus isolates are combined by cell type in S1 Fig. All of the ZIKV isolates tested, whether ZIKV^mam^ or ZIKV^mos^, replicated more quickly in Vero cells than in C6/36 cells (Fig 2). The contemporary isolates reached peak titers by 24 hpi, while ZIKV-MR-766^mam^ peaked at 2 dpi in Vero cells. All isolates reached a similar peak virus titer of 10^7^−10^8^ PFU/ml on Vero cells independent of host cell derivation (Fig 2 and S1 Fig), and the growth kinetics of ZIKV^mam^ and ZIKV^mos^ from an individual isolate closely mirrored each other (Fig 2), although ZIKV-PAN^mos^ and ZIKV-FLR^mos^ reached slightly lower titers than their mammalian-derived counterparts.

The one-step growth kinetics in C6/36 cells were similar between ZIKV^mam^ and ZIKV^mos^ isolates (Fig 2 and S1 Fig). We observed higher amounts of residual virus for ZIKV^mam^ compared to ZIKV^mos^ on C6/36 but not Vero cells; however, this did not affect when we first detected virus production in C6/36 cells (1 to 2 dpi). The kinetics of virus production between ZIKV^mam^ and ZIKV^mos^ stocks were nearly identical for each of the contemporary isolates (Fig 2), suggesting ZIKV^mam^ and ZIKV^mos^ have equal replicative ability in C6/36 cells. Peak titers on mammalian and mosquito cells were similar for the contemporary isolates, but virus titers peaked two to five days later on mosquito cells than on Vero cells (two day delay for ZIKV-PRV and ZIKV-FLR and five day delay for ZIKV-PAN). The peak titer of ZIKV-MR-766^mam^ in C6/36 cells was 30-fold lower than in Vero cells even with an additional five days of growth. Overall, these results suggest that the kinetics of a single round of replication of ZIKV are influenced primarily by the host cells rather than the deriving cells.

**Fig 3:**
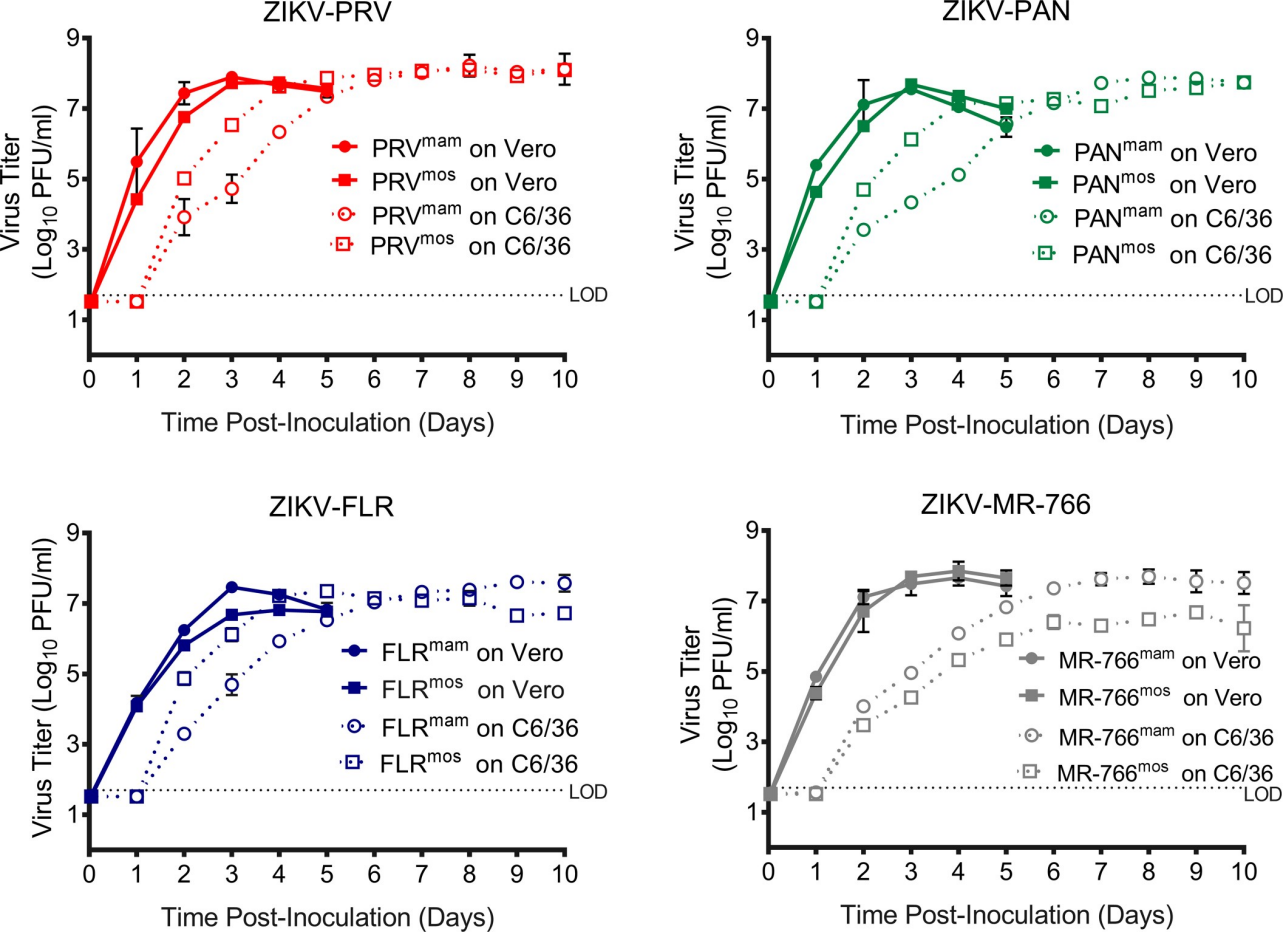
ZIKV^mam^ spread is reduced in C6/36 mosquito cells. Vero or C6/36 cells were inoculated at an MOI 0.1 with Vero mammalian cell-derived (ZIKV^mam^) or C6/36 mosquito cell-derived (ZIKV^mos^) ZIKV-PRV, ZIKV-PAN, ZIKV-FLR, and ZIKV-MR-766. Samples were collected at the indicated times and titered by plaque assay on Vero cells. Symbols represent the average, and error bars represent the standard deviation. Some error bars are smaller than the symbol and are not visible.

We next compared the ability of ZIKV^mam^ and ZIKV^mos^ to replicate and spread using a multi-step growth assay (Fig 3 and S2 Fig). Vero or C6/36 cells were infected at an MOI 0.005 with either ZIKV^mam^ or ZIKV^mos^. Samples were collected at various times after inoculation, and virus production was measured by PA. As observed in the one-step growth curves, viruses replicated more quickly in Vero cells than in C6/36 cells (Fig 3). For the three contemporary isolates, ZIKV^mos^ peak titers on Vero cells lagged behind ZIKV^mam^ peak titers by approximately one day. Titers of mosquito-derived and mammalian-derived virus peaked at similar levels on 3 dpi for ZIKV-PRV and ZIKV-PAN. ZIKV-FLR^mam^ and ZIKV-FLR^mos^ peaked on the same day (3 dpi), but ZIKV-FLR^mos^ titers were approximately 10-fold lower than ZIKV-FLR^mam^. Titers for ZIKV-MR-766^mam^ and ZIKV-MR-766^mos^ exhibited nearly identical growth on Vero cells. Peak titers on Vero cells were equivalent for ZIKV^mam^ and ZIKV^mos^ except for ZIKV-FLR^mos^, which had 5-fold lower peak titer than ZIKV-FLR^mam^. Multi-step growth on C6/36 cells revealed differences between the stocks. For the three contemporary isolates, ZIKV^mos^ reached peak virus titer two days earlier on C6/36 cells than ZIKV^mam^ (Fig 3 and S2 Fig). The prototype isolate, ZIKV-MR-766, which has been passed extensively in mammalian hosts and cells (Table 1), exhibited a different phenotype on C6/36 cells; peak titer for ZIKV-MR-766^mam^ was 10-fold greater than ZIKV-MR-766^mos^. In summary, the contemporary ZIKV isolates grew best on the type of cells from which the virus was derived (i.e. ZIKV^mam^ on Vero cells and ZIKV^mos^ on C6/36 cells), suggesting that low passage ZIKV isolates (Table 1) adapted in just one pass on either cell line.

### Stability of plaque phenotype

The plaque phenotypes (Fig 1) and the multi-step growth kinetics (Fig 3) suggest that adaptation through selection is occurring during cell culture passage. ZIKV-FLR^mos^ and ZIKV-FLR^mam^ had a particularly striking difference in plaque phenotype (Fig 1). The seed stock from BEI resources and ZIKV-FLR^mos^ stock produced plaques that were primarily pinprick-sized (Fig 1A), with occasional small or medium-sized plaques. In contrast, ZIKV-FLR^mam^ produced plaques ranging in size from tiny (< 1 mm in diameter) to large (> 4 mm in diameter). The large plaques had a ‘fuzzy’ phenotype, in which the plaque was not as pronounced against the neutral red-stained monolayer and the edges were less well defined. A subset of large plaques presented a ‘fried egg’ phenotype, where the center of the plaque was darker than the surrounding plaque. We examined when the large plaque phenotypes arose in the ZIKV-FLR^mam^ stock by conducting plaque assays on the daily samples collected during the initial virus amplification of the seed stock. Large plaques emerged in the ZIKV-FLR^mam^ stock on 3 dpi and became more prominent through harvest at 5 dpi. No large plaques were observed at any time through 6 dpi when ZIKV-FLR was grown on C6/36 cells.

**Fig 4:**
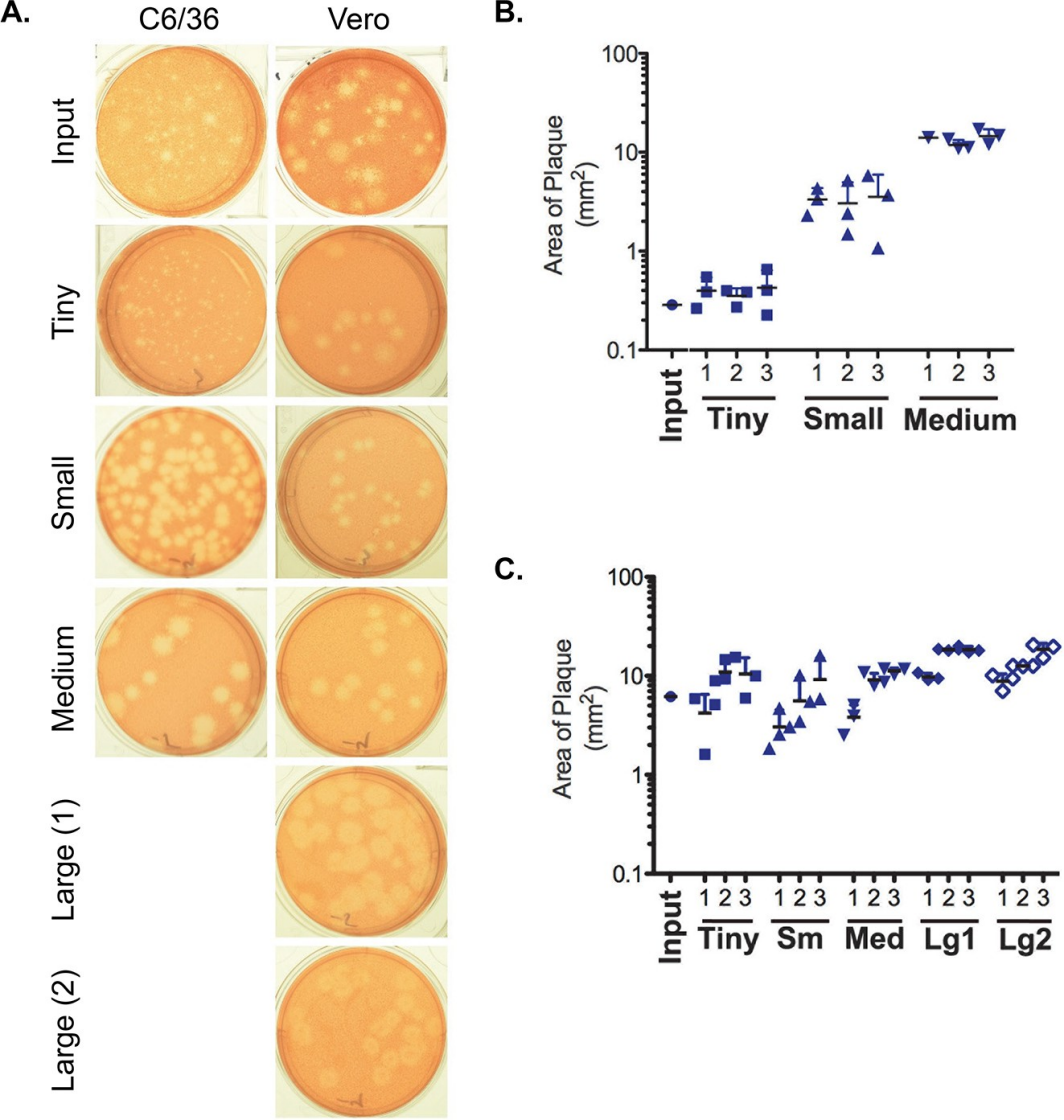
Plaque size is maintained in C6/36 cells, but not in Vero cells. ZIKV-FLR^mos^ was plaqued on Vero cells and representative tiny (L1), small (L2), or medium (L3) plaques were plaque purified over three rounds of passage through C6/36 cells. Similarly, ZIKV-FLR^mam^ was plaqued on Vero cells and representative tiny (L1), small (L2), medium (L3), large normal (L4) or large with a fried-egg phenotype (L5) plaques were plaque purified over three rounds of passage through Vero cells. Each passage of virus was titered on Vero cells. A representative of the final passage of each lineage is shown (A). The area of 30–50 plaques from each passage of each lineage derived from ZIKV-FLR^mos^ (B) and ZIKV-FLR^mam^ (C) was measured using ImageJ.

We examined the stability of the plaque phenotype by monitoring the phenotype over three rounds of plaque purification. Three replicate plaques from each size class for ZIKV-FLR^mos^ and ZIKV-FLR^mam^, were chosen for purification. ZIKV-FLR^mos^ had three size classes: tiny (lineage 1 (L1)), small (L2), and medium (L3). Plaques from ZIKV-FLR^mos^ were amplified on C6/36 cells for 7 days, and the supernatant was plaqued on Vero cells. This was repeated twice, for a total of 3 rounds of plaque purification. While the tiny and small plaques were abundant, only one medium-sized plaque was obtained in the first plaque pick round. Three plaques were picked in subsequent rounds. ZIKV-FLR^mam^ had four size classes: tiny (L1), small (L2), medium (L3), and large. Large plaques separated into two distinct phenotypes: fuzzy (L4) and fried-egg (L5). Plaques from ZIKV-FLR^mam^ were amplified on Vero cells for 3 days, and the supernatant was plaqued on Vero cells. This was repeated twice, for a total of 3 rounds of plaque purification. The area of 30–50 plaques for each plaque-purified biological clone was measured after each passage. The plaque phenotypes of the input population and the final passage from a representative clone of each lineage are shown in Fig 4A.

The plaque phenotype of viruses derived from and grown on mosquito cells remained consistent over three passages (Fig 4B). The average area of the plaques of the input ZIKV-FLR^mos^ was less than 1 mm^2^, representing the predominantly pinprick-sized plaques that comprise this population. ZIKV-FLR^mos^-L1 produced plaques similar in size to the average of the input virus. However, the plaque area of the ZIKV-FLR^mos^-L2 was about ten-fold larger than input, and the plaque area of ZIKV-FLR^mos^-L3 virus was almost 100-fold larger, demonstrating that the plaque size phenotype is maintained over passages in C6/36 mosquito cells.

ZIKV-FLR^mam^ plaques were larger and more heterogeneous than ZIKV-FLR^mos^. The average plaque area measured for ZIKV-FLR^mam^ of 13 mm^2^ was approximately 20-fold larger than for ZIKV-FLR^mos^. Furthermore, the plaques of ZIKV-FLR^mam^ lacked the size consistency seen in plaques of ZIKV-FLR^mos^. The size of plaques produced by each lineage of virus increased with passage number (Fig 4C). ZIKV-FLR^mam^-L1 began with tiny plaques of about 1 mm in diameter, but the average plaque size was larger than the average plaque size of the input population after two passages. The average plaque size for ZIKV-FLR^mam^-L2 and ZIKV-FLR^mam^-L3 also increased with passage, eventually surpassing the average plaque size of the input population. Plaques from the two large lineages, ZIKV-FLR^mam^-L4 and ZIKV-FLR^mam^-L5, also increased in size with passage although the initial plaque size was greater than the average plaque size of ZIKV-FLR^mam^. Both L4 and L5 lineages converged at a similar size, suggesting a maximum size limitation for the length of the PA. ZIKV-FLR^mam^-L5 originated from plaques with a fried egg phenotype. The proportion of plaques with this phenotype increased with passage; almost all plaques in the final passage had dark centers with lighter edges (Fig 4A). The stability of the plaque phenotypes differed on Vero or C6/36 cells. The overall trend toward increased plaque size after passage in Vero cells suggests the absence of a restriction that is present in C6/36 cells.

We examined the genomes of all lineages of the plaque purified pass 3 biological clones by next-generation sequencing as previously described [38]. The results of this analysis are shown in Table 3. We identified consensus level changes in all 10 gene products in Vero plaque-purified ZIKV and in six gene products (C, prM, E, NS2a, NS4a, and NS5) in C6/36 plaque-purified ZIKV. The gene products with the most mutations were prM, E, and NS1 (6, 10 and 7 amino acid changes, respectively); NS1 mutations were only observed in Vero plaque-purified ZIKV. Most mutations occurred in only one of the three replicate biological clones tested for that condition; however, there were two lineages, Vero L5 and C6/36 L3, for which all three replicate biological clones contained the same mutation. All of the biological clones of the L5 lineage of Vero plaque-purified ZIKV contained a serine to arginine mutation in prM and a glutamate to glycine mutation in NS2b, and these mutations were unique to the Vero L5 lineage. All of the biological clones of the L3 lineage of C6/36 plaque-purified ZIKV contained an isoleucine to threonine mutation in the capsid gene and an aspartic acid to glutamic acid mutation in the envelope gene. The envelope mutation was unique to the C6/36 L3 lineage, but the capsid mutation was also identified in one clone of the L5 lineage of Vero plaque-purified ZIKV.

**Table 3:** Consensus mutations identified in plaque-purified ZIKV clones.

Cell	Plaque size (lineage)	Gene Product *									
		C	prM	E	NS1	NS2a	NS2b	NS3	NS4a	NS4b	NS5
**Vero**	Tiny (L1)	ND**	R238G (1/3)*** Y289H (1/3)	Q634R (1/3) L779F (1/3)	Y969H (1/3)	A1303V (1/3)	ND	E1675D (1/3) K2089R (1/3)	ND	E2290D (1/3)	A3266V (1/3)
	Small (L2)	T57M (1/3)	K226E (1/3)	H578R (1/3)	K1039Q (1/3) G1086E (1/3)	ND	ND	ND	ND	ND	ND
	Medium (L3)	ND	Y289H (1/3)	ND	E936D (1/3) L963I (1/3) G1086E (1/3)	ND	ND	K2089R (1/3)	ND	ND	ND
	Large (L4)	ND	R238G (1/3)	Y371H (1/3) L761S (1/3)	V928A (1/3) K1039T (1/3)	A1303V (1/3)	ND	ND	K2244R (1/3)	ND	ND
	Large (L5)	I80T (1/3)	**S273R (3/3)**	ND	ND	ND	**E1399G (3/3)**	ND	ND	ND	ND
**C6/36**	Tiny (L1)	ND	ND	L486H (1/3)	ND	ND	ND	ND	ND	ND	ND
	Small (L2)	ND	Y278C (1/3) S231P (1/3)	A519V (1/3) R573K (1/3) T605I (1/3)	ND	V1289A (1/3)	ND	ND	ND	ND	K2565M (1/3) I2933V (1/3)
	Medium (L3)	**I80T (3/3)**	ND	**D373E (3/3)**	ND	ND	ND	ND	A2122V (1/3)	ND	ND

*Gene product with mutation listed as amino acid position in polyprotein.

**ND = no consensus-level mutations detected

*** Number in parentheses following mutation indicates the number of clones positive for the mutation over the total number screened.

## Discussion

ZIKV has attracted international attention due to its unprecedented co-incidence with Guillain-Barre syndrome and microcephaly in infants in the outbreak in the Americas and Caribbean that began in Brazil in 2015. Scientists worldwide have begun investigating the pathology of ZIKV infection. However, limited attention has been paid to the phenotypic characterization of ZIKV isolates, including variations in stocks, growth characteristics, or quantitative assays. These aspects are critical for reproducible results within and between laboratories. In this study, we compared assays used to titer ZIKV and characterized the growth kinetics and plaque phenotypes of four readily available ZIKV isolates, including three contemporary isolates and the Ugandan prototype isolate.

ZIKV titers are measured by a number of assays in the literature, but how titers compare across assays is unknown. The use of different assays restricts the ability of researchers to compare results between laboratories and evaluate the scientific conclusions. For this reason we compared infectious virus titers by PA, TCID_50_, and FFA and viral RNA titers by qRT-PCR. The PA is routinely used [43–45] as it provides consistent results and information about plaque morphology. In our hands, ZIKV^mam^ generally had higher titers than ZIKV^mos^ by PA. On the other hand, the PA requires cell death for read-out, which limits the cell types that can be used in the assay. Specifically, C6/36 cells cannot be used to titer ZIKV by PA because they do not exhibit cell death and subsequent plaque formation [46]. TCID_50_ assays, which have been used to examine ZIKV infection in mosquitoes [47], also require cell death, but TCID_50_ assays use less culture medium, do not require any overlay medium, and are more amenable to high throughput in a 96-well format compared to PA. However, ZIKV titers in the TCID_50_ assay were up to 22-fold lower than PA titers, and extension of the incubation time did not increase assay sensitivity. Assay choice is based on experimental constraints, but future studies using the TCID_50_ assay should acknowledge its reduced sensitivity compared to the PA.

FFA has been used to measure levels of flaviviruses including ZIKV [46, 48, 49]. FFA is appealing because it is independent of cell death, meaning any number of cell lines can be used [46]. Its use is limited, however, by the availability and quality of the appropriate antibody necessary to detect viral antigen. The set-up time for the FFA is similar to the PA, but reading the assay requires more time than the PA. Using the FFA, we were able to compare titers of ZIKV^mam^ and ZIKV^mos^ stocks on mammalian and mosquito cells (Table 2). FFA titers on Vero cells closely mirrored PA titers, and the results suggested that ZIKV^mam^ stocks had similar titers to ZIKV^mos^ stocks. When stocks were titered by FFA on mosquito cells, ZIKV-MR-766^mam^ titers were ~20-fold higher than ZIKV-MR-766^mos^ titers although both stocks had higher titers on mosquito cells than on mammalian cells. A different trend was observed for the contemporary viruses. ZIKV titers were highest when deriving cell and titering cell type were matched (i.e. the titer of ZIKV^mos^ was higher on C6/36 cells than on Vero cells, and the titer of ZIKV^mam^ was higher on Vero cells than on C6/36 cells). ZIKV^mos^ titers tended to be higher than ZIKV^mam^ titers. These observations suggest that ZIKV is more efficient at infecting or replicating in cells that match the cells from which the virus was derived. It is unclear why this effect was apparent only for the contemporary isolates. It is possible viruses with a long history of passage in culture and in mice, such as ZIKV-MR-766 (Table 1), may lose the ability to quickly adapt to different cell types.

Many laboratories report ZIKV RNA levels rather than or in addition to infectious virus [17, 44, 50, 51]; this may be the most convenient read-out of infection, as it eliminates cell culture completely. We demonstrate here that the median GE:PFU ratio for ZIKV was 1-3x10^3^ (Table 2). This is the first description of how ZIKV RNA levels correspond to infectious virus particles. The ZIKV ratio is similar to reported ratios for the flaviviruses DENV and yellow fever virus, which have GE:PFU ratios of 1-5x10^3^ [52, 53], but is approximately 10-fold higher than WNV [54–56]. The GE:PFU ratio of ZIKV^mam^ was 2–7 fold higher than ZIKV^mos^, similar to the difference observed between mammalian cell- and mosquito cell-produced WNV [55]. The data suggest that more non-infectious or immature particles may be produced during ZIKV infection of mammalian cells than mosquito cells and may indicate tighter regulation of infection in mosquito cells. We did not RNase-treat virus supernatant, so we cannot exclude the possibility that ZIKV-infected mammalian cells release more RNA into the supernatant than infected mosquito cells. Whether mammalian and mosquito cells differ in particle production or RNA release, disregarding the differences in GE:PFU ratio between host cells can lead to mistaken results. Some laboratories report RNA levels as infectious titer equivalents based on a standard curve. The standard curve, and therefore the extrapolated data, will be greatly influenced by the host cell used to generate the standard curve samples; for example, titers of mosquito cell-derived samples will appear lower when compared to a mammalian cell-derived standard curve than when compared to a mosquito cell-derived standard curve. Also, the difference in ratios should be kept in mind when comparing virus levels between species. A titer of 10^3^ genomic equivalents from a mammalian cell sample may not be equivalent to the same titer in a mosquito cell sample. Thus, the GE:PFU ratio should be taken into consideration when analyzing virus RNA levels.

We observed differences in the length of time peak virus titers were sustained in Vero and C6/36 cells. ZIKV titers remained fairly constant up to 10 dpi on C6/36 cells, while titers from Vero cells decreased by 3–4 dpi. This is likely due to differences in growth temperature; growth at 37°C may accelerate virus degradation compared to 28°C. The differences in titers may also reflect disparities in production of virus. C6/36 cells do not demonstrate CPE, and may produce virus longer than Vero cells, which succumb to infection. Similarly, C6/36 cells demonstrate sustained production of subgenomic replicon particles for up to 10 days after transfection [54]. The disparity in maintaining high levels of virus may reflect host physiological differences with important implications on flavivirus transmission.

We observed differences in the plaque phenotype of the ZIKV isolates that we tested. Previously reported differences in plaque phenotypes of ZIKV isolates have been correlated with ZIKV lineage. The ZIKV Asian lineage isolates tested by Willard et al. [42] produced larger plaques with less distinct borders compared to distinct, well-defined plaques of varying sizes from African lineage isolates. In contrast, the African isolate tested by Smith et al. [57] produced large plaques while the Asian lineage isolates produced very small plaques. While we also examined the prototype African lineage isolate (MR-766) [6] and contemporary Asian lineage isolates [58], we attributed plaque differences to host rather than lineage. Viruses grown in mammalian cells produced larger and more heterogeneous plaques while virus grown in mosquito cells produced smaller, more homogeneous plaques. We, therefore, hypothesize that the differences observed are due to host factors or constraints rather than virus lineage. The dichotomy was less striking for viruses previously passed in mammalian cells or animals (Fig 1, Table 1), which may explain the discrepancy in published results. Smith et al., whose findings are similar to those reported here, used low passage virus isolates, but the African isolate had been passed through mammalian cells five times versus a single passage for the Asian isolates [57]. The passage history of the isolates used by Willard et al. was not reported, but the Asian isolates had been passed fewer times (<10) than the African isolates (>25–100) [42]. The plaque phenotypic difference in stocks grown in mammalian and mosquito cells was most striking for ZIKV-FLR; the ZIKV-FLR^mos^ stock only produced very tiny, pin-prick-sized plaques compared to a larger and mixed plaque population for ZIKV-FLR^mam^ stock. Since ZIKV-FLR had been passed only in insect cells since its isolation, we posited that the different plaque sizes of the two stocks represented adaptation to growth in mammalian cells. In support of this hypothesis, we tracked the emergence of large plaques, never observed in ZIKV-FLR^mos^ stocks, to day 3 of the initial amplification of ZIKV-FLR on Vero cells. Furthermore, plaques isolated from ZIKV-FLR^mos^ passed true to the original size phenotype while ZIKV-FLR^mam^ plaques became increasingly large (Fig 4). Alternating growth on mosquito cells and plaquing on mammalian cells for the plaques isolated from ZIKV-FLR^mos^ may have constrained their adaptation. In contrast, plaques isolated from ZIKV-FLR^mam^ were grown and plaqued only on mammalian cells. The overall trend toward increased plaque size with passage in mammalian cells suggests loss of an insect cell adaptation and/or adaptation to mammalian cells in the absence of a restriction that is present in mosquito cells.

We sequenced the genomes of the plaque-purified viruses and identified consensus mutations scattered throughout the virus genome (Table 3). We found a greater number of mutations in the biological clones from the Vero cell-derived viruses than from the C6/36 cell-derived virus, consistent with work by others showing that genetic mutations accumulate more quickly when arboviruses are passed in mammalian cells compared to insect cells [59]. All three biological clones from the L5 (large) lineage of the Vero cell-derived viruses shared the same prM and NS2b mutations. The three biological clones from the L3 (medium) lineage of the C6/36 cell-derived virus contained the same mutations in the capsid and envelope genes. These are novel mutations, and the influence of these mutations on *in vitro* and *in vivo* virus replication is the subject of ongoing studies in our laboratory.

Disparity in initial infection may alternatively represent biochemical differences in the virus particle rather than genetic changes. For example, plasma membrane composition and protein glycosylation differences between mammalian and insect cells impact virus infectivity. Virus produced from insect cells contains less cholesterol than virus produced from mammalian cells [60], and lower levels of virion cholesterol reduces DENV infectivity [61]. Glycosylations of insect cell proteins are typically simpler, less branched, and generally not terminally sialydated compared to mammalian cell glycosylations [62], which can affect protein recognition and receptor binding. These differences are reflected in the resulting virus, and changes in both parameters have been shown to affect arbovirus infectivity [60, 63]. Previous studies by our laboratory have demonstrated delayed spread of insect cell-derived flavivirus during *in vivo* infection [54, 55]. Researchers should carefully consider which type of virus to use in an animal model, as mammalian cell-derived virus could produce misleading information compared to the more physiologically relevant mosquito cell-derived virus.

In conclusion, we characterized the growth kinetics of mammalian cell-derived and insect-cell derived ZIKV. Substantial differences exist between the contemporary isolates and the prototypic reference isolate ZIKV-MR-766, justifying the use of contemporary isolates to investigate ZIKV pathogenicity. In addition, insect cells appear more restrictive to ZIKV infection than mammalian cells, as demonstrated by the plaque size constraint of resulting viruses and the growth delay observed when ZIKV^mam^ infected insect cells. We identified four novel mutations associated with plaque size; how these mutations affect viral replication and virulence is under investigation by our laboratory. These results provide a foundation to investigate the underlying causes of ZIKV-induced pathology.

## Supporting information

S1 Fig Replication kinetics of single-round infection on Vero or C6/36 cells is similar between ZIKV^mam^ and ZIKV^mos^. Vero or C6/36 cells were inoculated at an MOI 1–3 with ZIKV-PRV, ZIKV-PAN, ZIKV-FLR, and ZIKV-MR-766 grown on either Vero mammalian cells (ZIKV^mam^) or C6/36 mosquito cells (ZIKV^mos^). ZIKV-MR-766^mos^ was omitted due to insufficient titer. Samples were collected at the indicated times and titered by plaque assay on Vero cells. Note: this is the same data as presented in Fig 2, but it is provided in an alternative layout to facilitate comparison between virus isolates.
(TIF)

S2 Fig ZIKV^mam^ spread is reduced in C6/36 mosquito cells. Vero or C6/36 cells were inoculated at an MOI 0.1 with Vero mammalian cell-derived (ZIKV^mam^) or C6/36 mosquito cell-derived (ZIKV^mos^) ZIKV-PRV, ZIKV-PAN, ZIKV-FLR, and ZIKV-MR-766. Samples were collected at the indicated times and titered by plaque assay on Vero cells. Note: this is the same data as presented in Fig 3, but it is provided in an alternative layout to facilitate comparison between virus isolates.
(TIF)
