# Supplementary figures and images for: Growth and adaptation of Zika virus in mammalian and mosquito cells
Source: PLoS Negl Trop Dis. 2018 Nov 12;12(11):e0006880. doi: 10.1371/journal.pntd.0006880 (PMC6258428; doi:10.1371/journal.pntd.0006880)

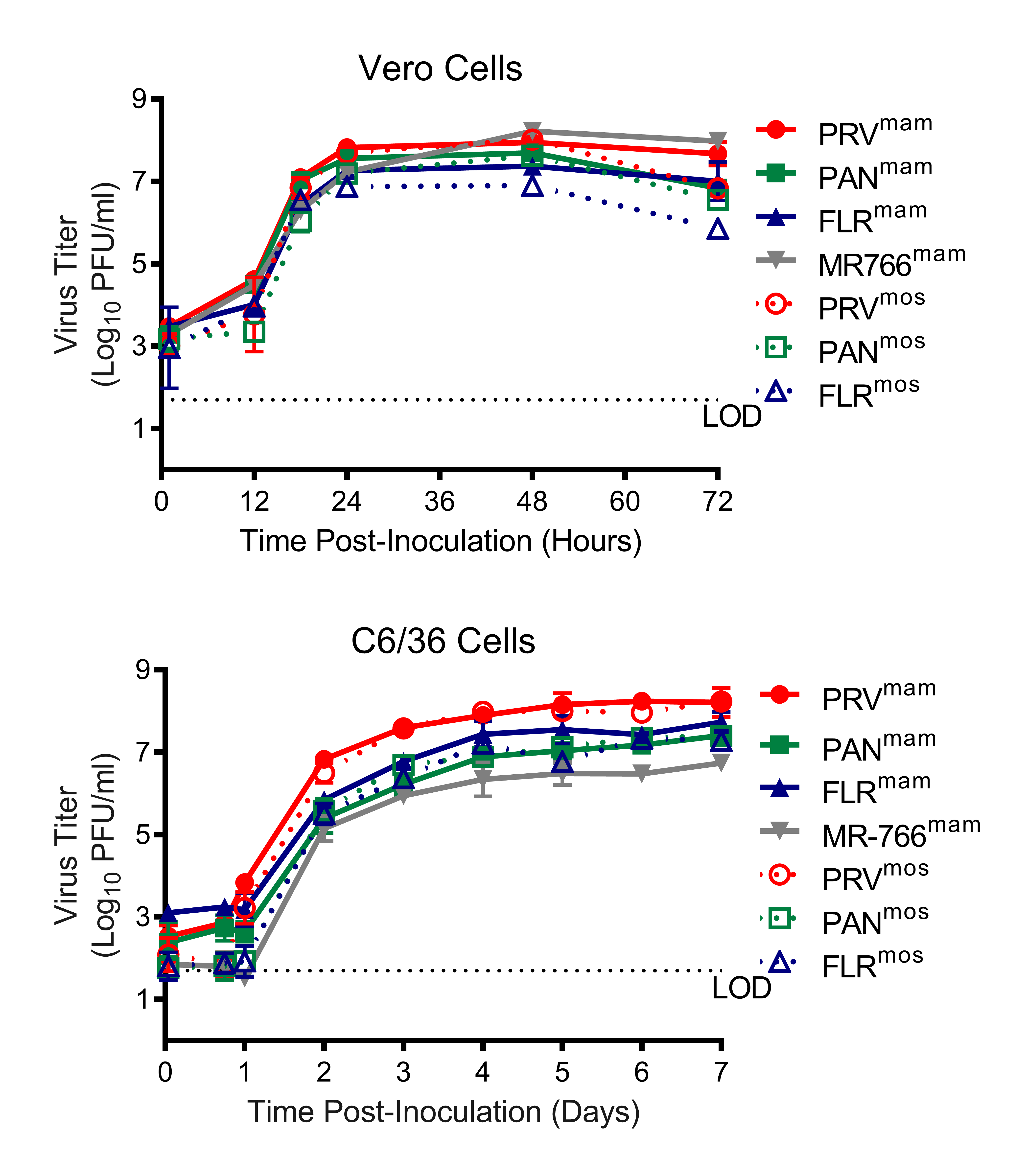

Supplement: S1 Fig — Vero or C6/36 cells were inoculated at an MOI 1–3 with ZIKV-PRV, ZIKV-PAN, ZIKV-FLR, and ZIKV-MR-766 grown on either Vero mammalian cells (ZIKVmam) or C6/36 mosquito cells (ZIKVmos). ZIKV-MR-766mos was omitted due to insufficient titer. Samples were collected at the indicated times and titered by plaque assay on Vero cells. Note: this is the same data as presented in Fig 2, but it is provided in an alternative layout to facilitate comparison between virus isolates. (TIF) [file pntd.0006880.s001.tif]

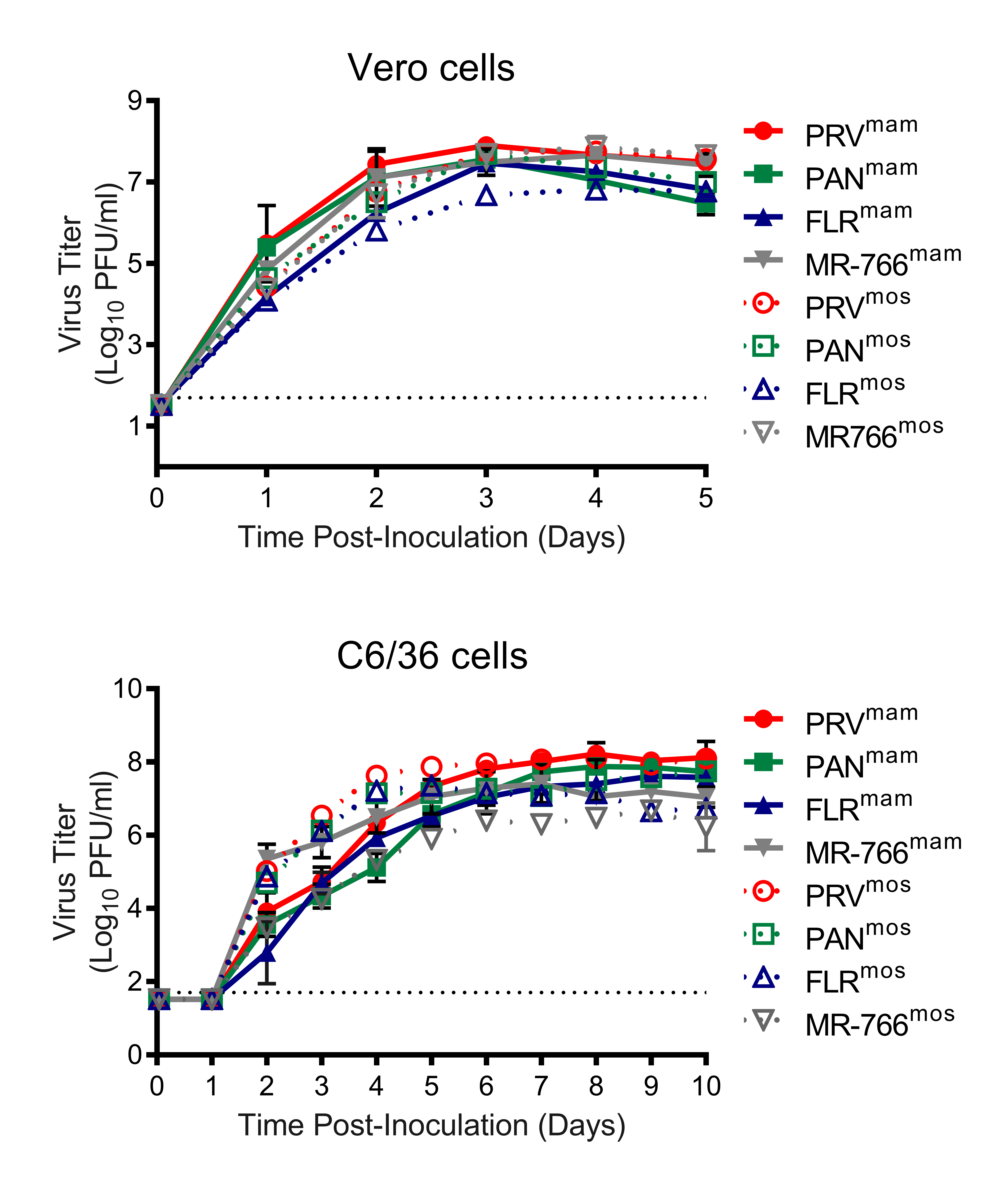

Supplement: S2 Fig — Vero or C6/36 cells were inoculated at an MOI 0.1 with Vero mammalian cell-derived (ZIKVmam) or C6/36 mosquito cell-derived (ZIKVmos) ZIKV-PRV, ZIKV-PAN, ZIKV-FLR, and ZIKV-MR-766. Samples were collected at the indicated times and titered by plaque assay on Vero cells. Note: this is the same data as presented in Fig 3, but it is provided in an alternative layout to facilitate comparison between virus isolates. (TIF) [file pntd.0006880.s002.tif]
